# Epidemiological, Morphological, and Morphometric Study on *Haemonchus* spp. Recovered From Goats in Egypt

**DOI:** 10.3389/fvets.2021.705619

**Published:** 2021-10-26

**Authors:** Ahmed Gareh, Nagwa M. Elhawary, Amin Tahoun, Amany M. Ramez, Dina M. M. EL-shewehy, Elzahara Elbaz, Marwa I. Khalifa, Khalaf F. Alsharif, Refaat M. A. Khalifa, Ahmed K. Dyab, Mohmed Elsalahy M. Monib, Mohsen I. Arafa, Ehab Kotb Elmahallawy

**Affiliations:** ^1^Department of Parasitology, Faculty of Veterinary Medicine, Aswan University, Aswan, Egypt; ^2^Department of Parasitology, Faculty of Veterinary Medicine, Kafrelsheikh University, Kafr El Sheikh, Egypt; ^3^Department of Animal Medicine, Faculty of Veterinary Medicine, Kafrelshkh University, Kafr El Sheikh, Egypt; ^4^Zoology Department, Faculty of Science, Mansoura University, Mansoura, Egypt; ^5^Department of Internal Medicine and Infectious Diseases, Faculty of Veterinary Medicine, Mansoura University, Mansoura, Egypt; ^6^Department of Food Hygiene, Faculty of Veterinary Medicine, Aswan University, Aswan, Egypt; ^7^Department of Clinical Laboratory Sciences, College of Applied Medical Sciences, Taif University, Taif, Saudi Arabia; ^8^Department of Parasitology, Faculty of Medicine, Assiut University, Assiut, Egypt; ^9^Department of Parasitology, Animal Health Research Institute, Assiut, Egypt; ^10^Department of Zoonoses, Faculty of Veterinary Medicine, Sohag University, Sohag, Egypt

**Keywords:** epidemiology, morphology, *Haemonchus* spp., scanning electron microscope (SEM), goats, Egypt

## Abstract

Goats can be infected by multiple groups of external and internal parasites. *Haemonchus* spp. are among abomasal parasites that can result in higher mortality and several considerable economic losses in goats. Early detection of parasites and better understanding of the major risk factors associated with infection are among the main strategies for controlling the infection. Considering this, information on hemonchosis in goats from Egypt, and the contribution of goats in the maintenance of the epidemiological foci of the disease is limited. This study investigated the prevalence of *Haemonchus* species among 240 abomasum samples collected during postmortem examination of goat carcasses from Assiut Governorate, Egypt. Moreover, the association of the major risk factors to describe the epidemiological pattern of the disease was explored. This study demonstrated that 16.66% of abomasa samples harbored *Haemonchus* species. Additionally, age, sex, and sampling season were the most significant risk factors associated with infection. Following the variable factors under study, goats aged 1 year or older were at higher risk, with an infection rate of 22.14% (31 of 140), than those younger than 1 year (9%) [*p* = 0.008; odds ratio (OR) = 2.87; 95% confidence interval (CI), 1.30–6.35]. The infection rate was 25% (19 of 76) in males and 12.8% (21 of 164) in females [*p* = 0.024; odds ratio (OR) = 2.26; 95% confidence interval (CI), 1.13–4.53]. Moreover, the exposure to infection was higher in summer (22.22%) than in winter (8.33%) (*p* = 0.007; odds ratio (OR) = 0.318; 95% confidence interval (CI), 0.139–0.725). More importantly, three species of the parasite—*Haemonchus contortus, Haemonchus placei*, and *Haemonchus longistipes*—were identified for the first time, and the confirmation of the identification and morphological characterization of the worms was performed using light microscopy and SEM. Collectively, this study reveals interesting epidemiological, morphological, and morphometric findings associated with the occurrence of hemonchosis among goats in Egypt. This study suggests further research for exploring the major circulating species of the parasite in Egypt, which is mandatory for controlling the disease.

## Introduction

*Haemonchus* or barber's pole worm is a major abomasal parasite of ruminants with a global distribution (1, 2). Approximately 12 species were identified within the same genus in domestic ruminants (3). Among others, *Haemonchus contortus* is considered the most economically important gastrointestinal nematode in goats in tropical and subtropical regions (4, 5). However, several studies have revealed that other species, for example, *Haemonchus placei* and *Haemonchus similis*, are also among the most pathogenic nematodes in goats worldwide (6). Importantly, these parasite species result in significant production losses due to morbidity, mortality, and cost of treatment (6). According to their clinical impacts, anemia, digestion–absorption syndromes, and weight loss are among the clinical signs of hemonchosis (7).

According to their life cycle, *Haemonchus* spp. have a direct cycle that involves pre-parasitic (free living stages) and parasitic stages. Male and female adults inhibit the abomasum or small intestine. Clearly, worms can be found attached to the mucosa or free in the lumen (8, 9).

According to their morphology, several parameters and structures have been used to study the morphology of male *Haemonchus* worms, such as body length, cervical papillae length, spicule length, gubernaculum length, barb length (distance from tip to hook), and the number of cuticular ridges. Female *Haemonchus* worms are 18–30-mm long and are easily recognized by the “barber's pole” appearance of the white ovaries and uteri twisting for the length of the worm around a red blood-filled intestine, whereas males are 10–20-mm long and uniformly reddish-brown (10). The body length, cervical papillae length, number of cuticular ridges, and vulva flap morphology represent the main criteria for identifying and differentiating female parasite species (11). Additionally, *Haemonchus* has a tooth or lancet in its poorly developed oral cavity, which helps perforate the gastric mucosa and suck blood (12). Therefore, both the L4 larval stage and the adult worms of *Haemonchus* spp. might cause punctiform hemorrhages at feeding sites on the abomasal mucosa (10).

The diagnosis of hemonchosis could be performed using various methods, for example, direct fecal smears, fecal flotation for detecting parasitic eggs, and postmortem examination for determining immature and adult worms (4). Furthermore, information on the role of scanning electron microscopy (SEM) in differentiating various *Haemonchus* spp. in goats from Egypt is limited due to financial constraints (13). Thus, hemonchosis in goats in Egypt is complicated by the scarcity of available data and unauthorized slaughtering of goats in private slaughter houses. Given the aforementioned information, this study was designed to investigate the overall prevalence and the major risk factors associated with the occurrence of *Haemonchus* spp. recovered from the abomasa of goats in Assiut Governorate, Egypt. Moreover, this study provided a descriptive morphological and morphometric identification and differentiation of the recovered parasite species.

## Materials and Methods

### Ethical Considerations

Ethical approval was obtained from a guidance of Research, Publication, and Ethics of the Faculty of Veterinary Medicine, Kafrelsheikh University, Egypt, which complied with all relevant Egyptian laws in research and publication. The ethical approval number is KFS-2018/3.

### Study Area and Sample Collection

This study targeted morphological and morphometric differentiation of *Haemonchus* spp. in Baladi goats (native breed) admitted to Beni Adi and Manfalout private abattoirs, Assiut Governorate, Egypt, from May 2018 to March 2020. In this study, 240 abomasa specimens were investigated by visual inspection. The abomasal specimens were then transported to the laboratory of the Department of Medical Parasitology, Faculty of Medicine, Assiut University, Egypt, for further examination. The clinical signs that appeared on the goats under study before slaughtering and during postmortem examination were recorded. Data on the age and sex of the animals under study were registered. Additionally, sampling was conducted during different seasons to record the seasonal variations of the disease in goats. Therefore, 96 goat carcasses were examined in cold seasons (from December to March), and 144 carcasses were examined in hot seasons (from June to September).

### Preparation and Examination of Samples

Samples were prepared and examined according to previously described protocols (14, 15). Briefly, after slaughtering, the abomasum was removed and separated from other compartments, ligated at both ends, and transferred directly to the laboratory in sterile labeled plastic bags. Each abomasum was opened along its greater curvature using a pair of scissors, and the contents were poured in a glass beaker and were then processed by repeated washings, sedimentation, and decantation until the supernatant was clear enough for easier worm counting. The abomasum and its contents were checked carefully (15, 16). Each adult worm was collected and checked under a dissecting microscope. The worms were also flushed with tap water to remove food residues.

### Fixation and Preservation of Nematodes

The collected worms were immersed in 70% alcohol at 60°C, then kept into sterile bottles containing 70% alcohol and 5% glycerin, and cleared in lactophenol for 24 h. The specimens were mounted in Canada balsam within a glass slide, covered with a cover slide, and left in an oven at 37°C to dry as previously described (17). The *Haemonchus* spp. collected were morphologically identified based on a previously described protocol (18).

### Morphological and Morphometric Examination of *Haemonchus* spp

The protocol of processing and preparation of the abomasal samples collected for the examination of *Haemonchus* spp. was performed as described elsewhere (19). Briefly, the specimens were transported to the Electron Microscopy Unit, Assiut University, and washed in saline to remove any stocked particles, while the fixation was performed in 5% glutaraldehyde for 24–72 h. Then, the specimens were washed in sodium cacodylate buffer (pH 7.3) four times for 15 min each, followed by fixation for 2 h by adding 1% osmium tetroxide. The samples were washed again with sodium cacodylate buffer (pH 7.3) three times, and the dehydration was performed in ascending concentrations of ethanol (i.e., 30, 50, 70, and 90%, in this order). This step was followed by a double change of absolute ethanol for 24–48 h; then, the specimens were cleared in xylene overnight and air dried. The samples were then incubated at 20–25°C, stocked in a double Scotch tape carbon, and coated with gold. Moreover, the specimens were examined using SEM for detecting *Haemonchus* spp. and morphometric analysis (Joel, JSM-5400LV Scanning Electron Microscope, Tokyo 1993, Japan). For the identification of nematodes, the specimens were morphologically examined for different aspects, that is, length, whereas their width and diameter were measured using the measure interactive function, according to the protocols described elsewhere (1, 20, 21).

### Statistical Analysis

In this study, Fisher's exact test [with 95% confidence interval (CI)] was used to analyze the data, mainly the impact of different individual variable factors (i.e., sex, age, and season) on the prevalence of disease in goats. Data were computed using GraphPad Prism, and *p* < 0.05 were used to denote statistical significance.

## Results

### Epidemiological Data and Clinical Manifestations

**Figure 1:**
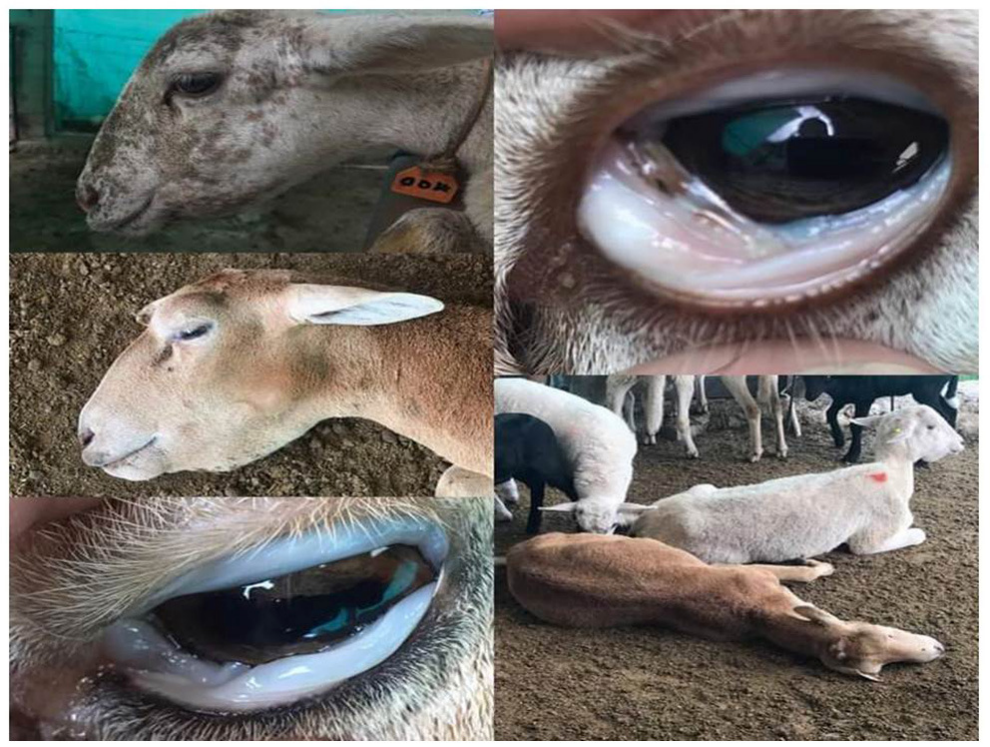
Clinical signs appeared on examined animals including pale conjunctiva, bottle jaw, weakness, and debilitation.

**Figure 2:**
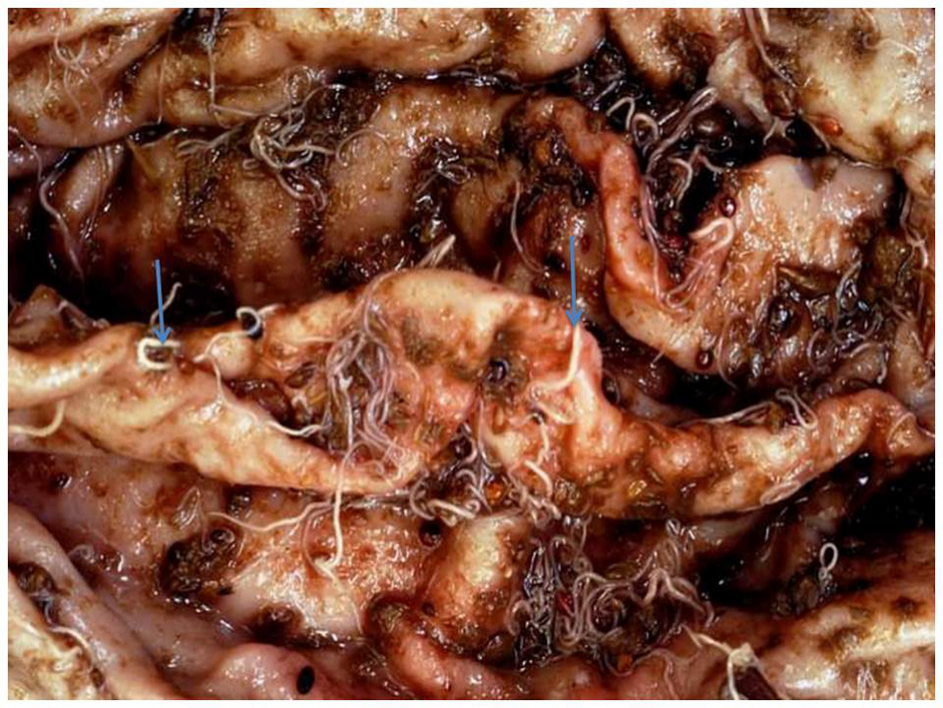
Macroscopic appearance of abomasa following postmortem examination with adult worm.

The overall prevalence of *Haemonchus* spp. in the examined slaughtered goats was 16.66% (40 of 240). The infected animals also showed various clinical signs, including diarrhea, weakness and debilitation, anemia, and weight loss (Figure 1). Some goats (n = 24) showed submandibular edema (bottle jaw). Some animals (n = 24) experienced hemorrhage on the mucosa of the abomasa, and worms were detected during postmortem examination (Figure 2). Furthermore, the occurrence of hemonchosis in the examined animals was significantly affected by the age and sex of the animals and season. Goats aged 1 year or older were at a higher risk than those younger than 1 year [odds ratio (OR) = 2.87; 95% CI, 1.30–6.35]. Regarding sex, males were more affected by hemonchosis than females (OR = 2.26; 95% CI, 1.13–4.53). Moreover, the seasonal variability of the disease was observed, and the animals were at a higher risk in summer than in winter (Table 1).

**Table 1:** Risk factors and prevalence of hemonchosis in goats after adjusting for age, sex, and season.

**Variable**	**No. examined**	**Positive no. (%)**	**Negative no. (%)**	**Odds ratio (95% CI), *p*-value**
**Age**				
≤1 year	100	9 (9)	91 (91)	2.87 (1.30–6.35) *p =* 0.008
>1 year	140	31 (22.14)	109 (77.85)	
**Sex**				
Female	164	21 (12.80)	143 (87.19)	2.26 (1.13–4.53) *p =* 0.024
Male	76	19 (25)	57 (75)	
**Season**				
Summer	144	32 (22.22)	112 (77.77)	0.318 (0.139–0.725) *p =* 0.007
Winter	96	8 (8.33)	88 (91.66)	
Total	240	40 (16.66)	200 (83.33)	

### Morphological Structure of the Recovered *Haemonchus* spp

In this study, *Haemonchus* spp. were recovered from 40 (16.66%) of the 240 abomasa examined. Macroscopically, the worms were bright red. According to their dimensions, males measured 10–20 mm in length and 0.3–0.4 mm in width, whereas females measured 18–30 mm in length and 0.3–0.4 mm in width.

### Microscopic Appearance of the Identified *Haemonchus* spp

**Figure 3:**
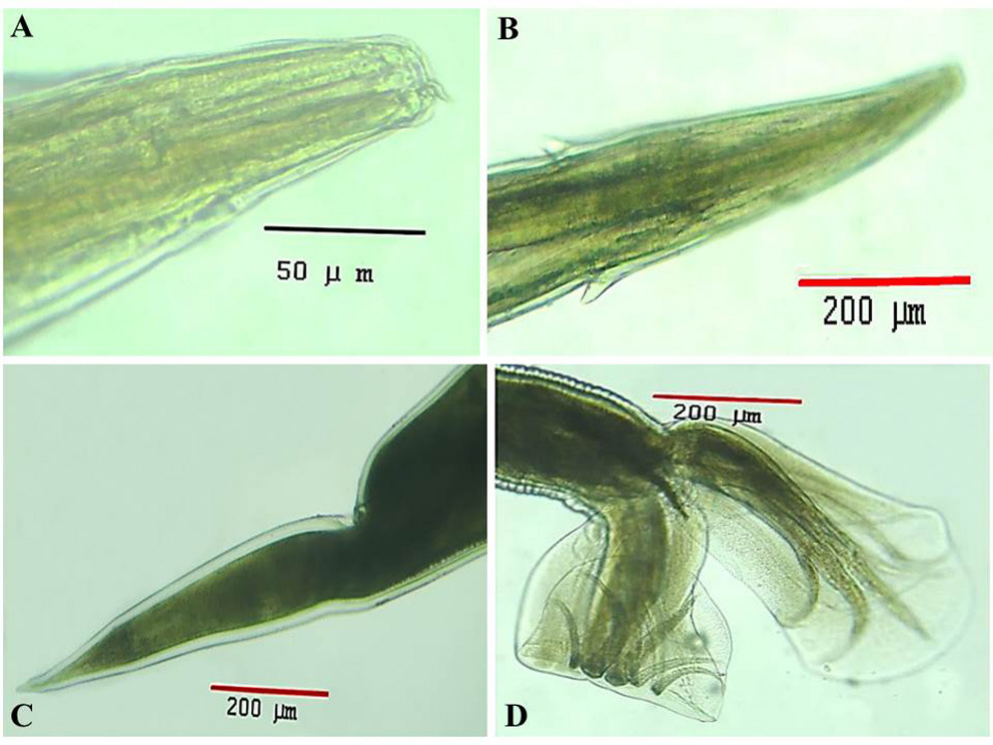
Morphological and microscopic appearance of *Haemonchus* spp. in goats. **(A)** The anterior end of *Haemonchus* spp. showing the buccal capsule containing a lancet (arrow). **(B)** The anterior end of *Haemonchus* spp. showing a cervical papilla-like spine (arrow). **(C)** The tail region of female *Haemonchus* spp. showing an anal pore (arrow). **(D)** The posterior end of male *Haemonchus* spp. showing a copulatory bursa.

**Figure 4:**
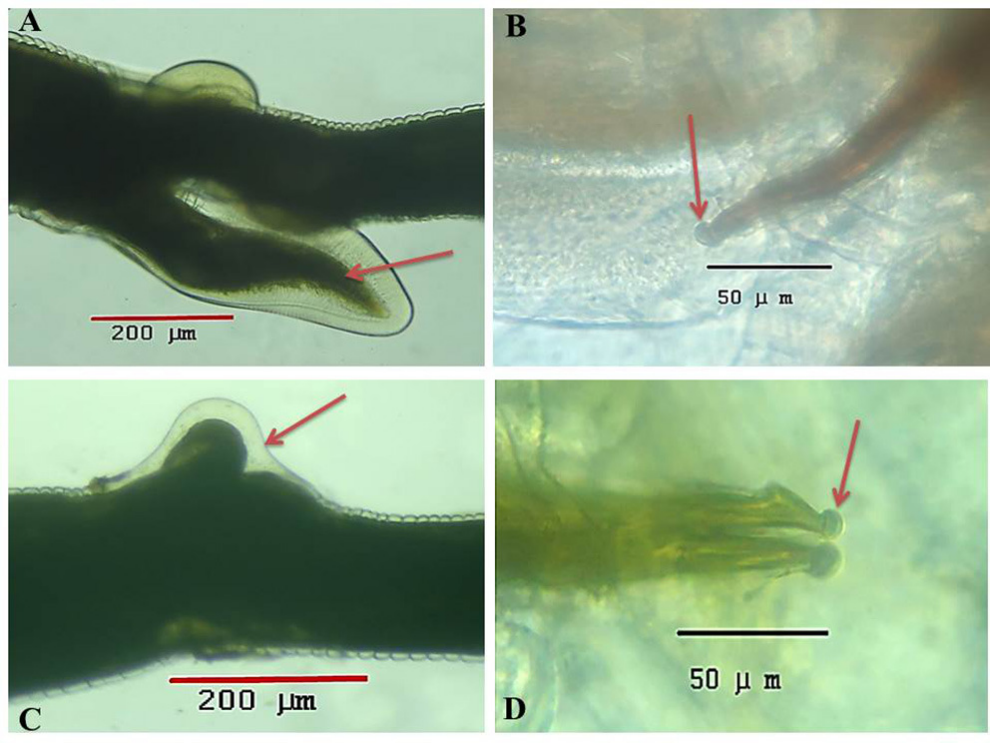
Microscopic appearance of *Haemonchus* spp. isolated from the abomasum of goats using a light microscope. **(A)** The vulvar region of female *Haemonchus contortus* showing a thumb-like vulvar flap (arrow). **(B)** Higher magnification of the spicules of male *H. contortus* showing fused barbed tips (arrow). **(C)** The vulvar region of female *Haemonchus placei* showing a reduced knob-like vulvar flap (arrow). **(D)** Higher magnification of the spicules of male *H. placei* and *H. longistipes* showing split barbed tips (arrow).

Under a light microscope, the morphology of the *Haemonchus* nematodes under study identified the anterior end that showed a small buccal capsule containing a dorsal lancet (Figure 3A). Furthermore, a pair of wedge-shaped cervical papilla-like spines were located at a distance of 0.5 mm from the anterior end (Figure 3B), while the anal opening was located at a distance of 0.6–0.8 mm from the posterior end (Figure 3C). The bursa of male nematodes was well-developed with two lateral lobes and one asymmetrically placed dorsal lobe, which was situated against the left lateral lobe (Figure 3D). The vulva of female *Haemonchus* spp. was detected at a distance of 4–6 mm from the end of the tail and was covered by a vulvar flap, which is a large prominent thumb-like linguiform, predominating in *H. contortus* (Figure 4A). A higher magnification of *H. contortus* male spicules showed fused barbed tips (Figure 4B). Moreover, some specimens showed a reduced knob-like vulvar flap, predominating in *H. placei* (Figure 4C), and a higher magnification of *H. placei* and *H. longistipes* male spicules showed split barbed tips (Figure 4D).

### Scanning Electron Microscopy of *Haemonchus* spp

**Figure 5:**
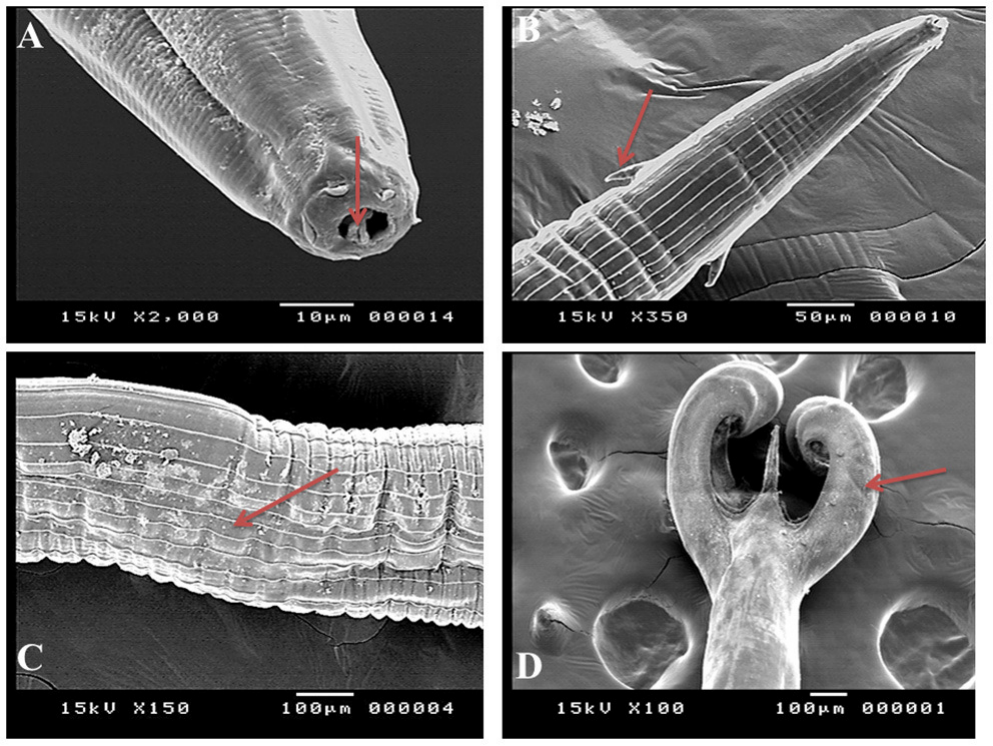
Morphometric appearance of *Haemonchus* spp. isolated from the abomasum of goats using scanning electron microscopy. **(A)** *Haemonchus* spp. showing the buccal capsule containing a lancet (arrow). **(B)** *Haemonchus* spp. showing a cervical papilla-like spine (arrow). **(C)** *Haemonchus* spp. showing longitudinal cuticular ridges (arrow). **(D)** The tail region of male *Haemonchus* spp. showing a copulatory bursa (arrow).

**Figure 6:**
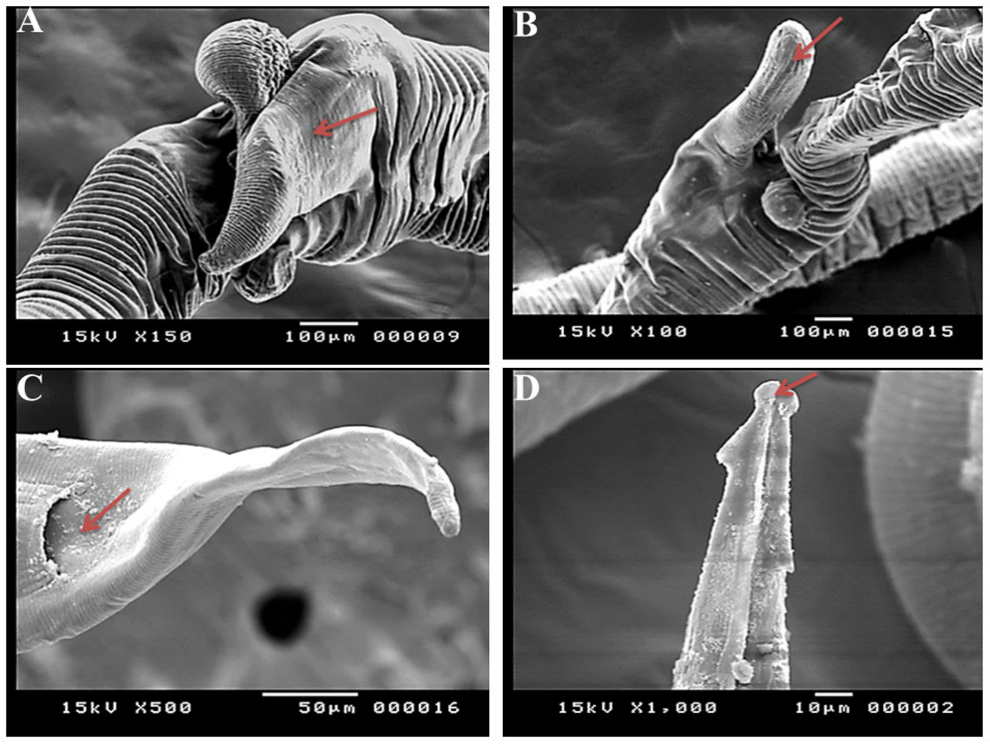
Morphometric appearance of *Haemonchus contortus* isolated from the abomasum of goats using scanning electron microscopy. **(A,B)** The vulvar region of female *H. contortus* showing a polymorphism vulvar flap (arrow). **(C)** The tail region of female *H. contortus* showing an anal pore (arrow). **(D)** The spicules of male *H. contortus* showing fused barbed tips (arrow).

**Figure 7:**
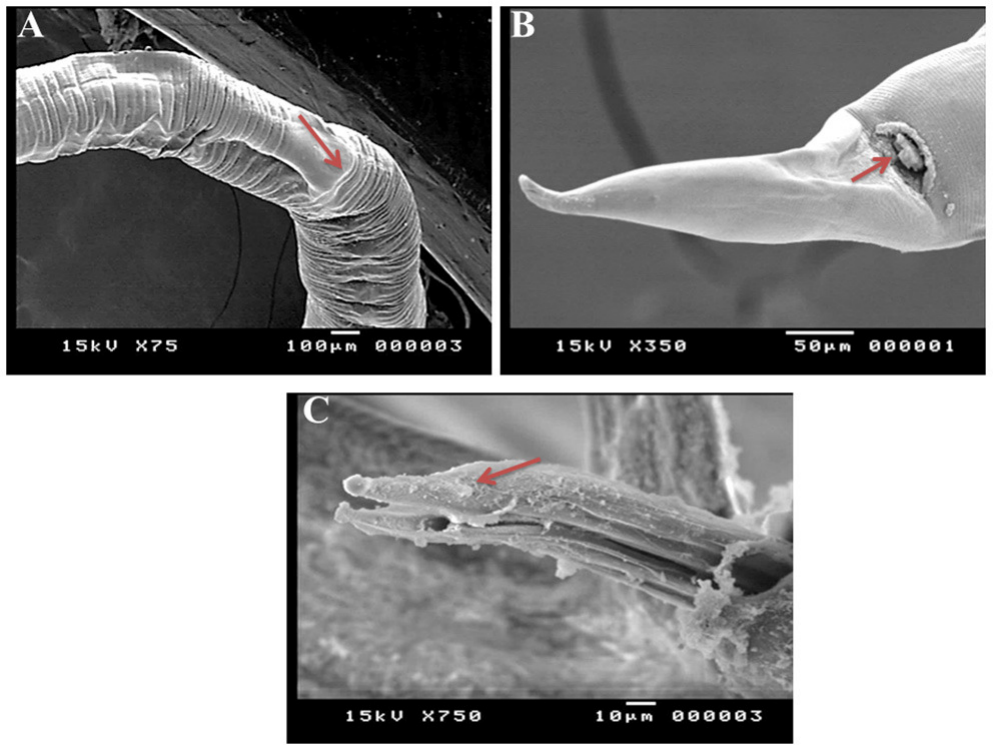
Morphometric appearance of *Haemonchus placei* isolated from the abomasum of goats using scanning electron microscopy. **(A)** The vulvar region of female *Haemonchus placei* showing a reduced knob-like vulvar flap (arrow). **(B)** The tail region of female *H. placei* showing an anal pore (arrow). **(C)** The spicules of male *H. placei* showing split barbed tips (arrow).

**Figure 8:**
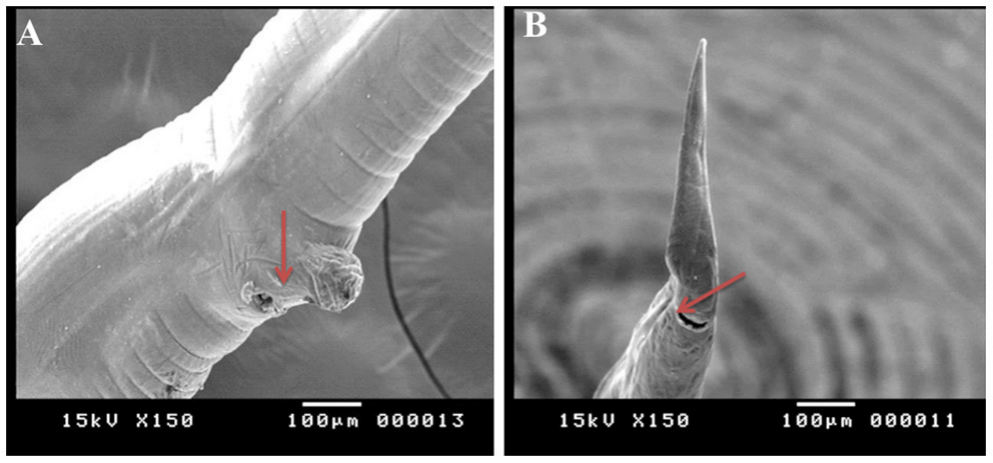
Morphometric appearance of *Haemonchus longistipes* isolated from the abomasum of goats using scanning electron microscopy. **(A)** The vulvar region of female *Haemonchus longistipes* showing a small rough nut-like vulvar flap (arrow). **(B)** The tail region of female *H. longistipes* showing an anal pore (arrow).

SEM identified three species of *Haemonchus*, namely, *H. contortus, H. placei*, and *H. longistipes*. The morphometric measurements of the identified *Haemonchus* spp. are shown in Table 2. All *Haemonchus* spp. showed similar cuticular ultrastructures, and the oral cavity had four lancets: two dorsoventral lancets and two lateral lancets (Figure 5A). The anterior end of the body was characterized by a transverse striated cuticle and longitudinal ridges. They also have two lateral disposed cervical spines, which were directed lateroposteriorly, measuring 40 microns each, with blunt ends (Figure 5B). The Cuticle is covered by longitudinal striations (Figure 5C). Alternatively, vulvar flaps were different in the detected species. In this regard, the vulvar flaps in *H. contortus* were thumb-like, adapted with longitudinal furrows, but sometimes showed polymorphism with a transverse striated surface (Figures 6A,B). The posterior end of female worms was smooth with tapering blunt ends, with a dome-like anal opening in *H. contortus* (Figure 6C) and *H. placei* (Figure 7B), whereas it appeared an elongated transverse slit in *H. longistripes* (Figure 8B). Alternatively, the vulvar flap in female *H. placei* was reduced to a small knob-like smooth flap (Figure 7A), and that in *H. longistipes* was small, rough, and nut-like in appearance (Figure 8A). The posterior end of male worms showed the typical umbrella-like appearance of the bursa, but the bursal rays could not be illustrated (Figure 5D). The spicules of male *H. contortus* showed fused barbed tips (Figure 6D), while those of male *H. placei* and *H. longistipes* had split barbed tips (Figure 7C).

**Table 2:** Morphometric measurements of the *Haemonchus* spp. recovered.

***Haemonchus* spp**.	***H. contortus***	***H. placei***	***H. longistipes***
**Female vulvar flap**			
Shape	Thumb like or linguiform with longitudinal furrows	Knob-like smooth flap	Small rough nut like
Length	260.23 ± 10.18 μm	81.87 ± 4.83 μm	62.63 ± 7.19 μm
**Male bursa spicule**			
Shape	Fused barbed tips, with two lateral papillae alongside the two spicule	Split barbed tips	Split barbed tips
Length	364.58 ± 12.46 μm	326.59 ± 11.61 μm	280.56 ± 12.68 μm
**Posterior end**			
Shape of anal pore	Dome like	Dome like	Elongated transverse slit
Tail length	312.5 ± 5.8 μm	279.05 ± 9.97 μm	179.01 ± 9.96 μm

## Discussion

Goats can be parasitized by several external and internal parasites (22–24). Among others, *Haemonchus* is considered one of the most common parasites in goats, sheep, and cattle worldwide (3, 25–27). Several studies have revealed that sheep and goats are more susceptible to *Haemonchus* spp. than cattle (3). These parasites are blood suckers that might result in severe anemia, hypoproteinemia, intermandibular edema (bottle jaw), and death, resulting in several considerable economic losses (3). Clearly, the implementation of strict control strategies for combating this parasitic disease seems crucial (28). This study showed interesting data related to the occurrence of *Haemonchus* spp. recovered from the abomasum of goats in Egypt, along with the major risk factors associated with infection. Additionally, this study revealed the role of morphological and morphometric techniques for identifying and differentiating various *Haemonchus* spp. using light microscopy and SEM. Moreover, for the first time, this study showed the occurrence of *H. placei* and *H. longistipes* in goats from Assiut Governorate, Egypt. In this study, the overall prevalence of the identified *Haemonchus* spp. was 16.66% (40/240). Several studies have documented the high incidence of *H. contortus* in small ruminants from Egypt (29–31). Nearly similar results were reported by Elshahawy et al. (29) in Upper Egypt, who detected *H. contortus* in 15.5% of the goats examined. In contrast, the obtained results in this study were lower than those mentioned by Almalaik et al. (32) in South Darfur State in Western Sudan, who reported *H. contortus* in 26% of the animals examined. Furthermore, Arafa (33) in Beni Suef has found that 19.5% of the examined goats were infected with *H. contortus*. Additionally, Bibi et al. (34) have found that 50% of the goats under study have *H. contortus* in Pakistan, and Nahar et al. (35) have reported a prevalence rate of 57.8% for goats harboring *H. contortus* in Bangladesh. The results of this study are also lower than those mentioned by Gadahi et al. (36), who have revealed that the incidence of *Haemonchus* was 64.19% in goats in Rawalpindi and Islamabad (Pakistan). Moreover, a study in Rwanda has revealed that 71.8% of the goats examined harbored *H. contortus* (37). In contrast, our results are slightly higher than that reported in a study conducted in Western Chitwan of Nepal, where *H. contortus* was reported in 13.89% of the examined goats (38). Taken into account that most cases were kept indoor before slaughter, the low number of infected animals in the present study *vs*. those reported elsewhere might be attributed to the periodical and regular use of anthelminthics by the majority of the goats' owners, which showed various degree of effectiveness against *Haemonchus* spp. (39). Revising the available literature, lower levels of infection by *Haemonchus* spp. in small ruminants could be also associated with a series of clinical, signs such as severe chronic anemia, pale pink to white conjunctiva, bottle jaw, loss/poor appetite and weight loss, and hemorrhage on the mucosa of the abomasa during postmortem examination (40, 41), which are in harmony with those reported in our present study. More importantly, the variation in the prevalence rates could be also attributed to several factors, including the system of management and pasturing, level of education of the farmer, climatic variations, the number and size of the collected samples, management practices, the adopted control measures and eradication programs used in the country, the purpose of raising goats, feeding practices, the age of the goats, and deworming intervals (29, 42–45).

Among the analyzed variables in this study, age was a significant factor affecting hemonchosis in goats. Goats aged one year and older were more susceptible to infection and had greater odds of exposure than younger goats. Similarly, a study has reported prevalence rates of hemonchosis of 37.9 and 49% in young and adult animals, respectively (46). This study reported similar findings to those observed by Shankute et al. (47), who have shown a higher rate of hemonchosis in adult animals (86.9%) than younger animals (86.57%) (47). In contrast, the results of this study are different from those recorded in a previous study (48) in Sri Lanka, where the eggs of gastrointestinal nematodes were found in 89% of the kids, 94% of the young goats, and 84% of the adult goats, and the identification of gastrointestinal nematodes revealed *H. contortus* (90%). This difference might be attributed to several factors, including grazing habits, management practices and breeding systems of the examined animals, anthelmintics used, locality, phase of infection during the examination, detection method, and laboratory techniques used (43, 49–51). Another study has reported that animals below 1.6 years of age are more susceptible to parasitic infection than adult ones (52). This may be because with advancement of age, the vigor of animals becomes better, and they develop resistance to parasitic diseases (53).

In this study, sex was an epidemiological risk factor involved in the distribution of *Haemonchus* spp. Thus, the prevalence rate was higher in male goats (25%) than that in female goats (12.8%). Similar results were reported in previous studies (54), where more males were infected than females. In contrast, some studies have reported a higher occurrence of these parasites in females than in males (55), whereas others have reported that sex or age has no significant influences on the occurrence of the parasites in small ruminants (50). This difference might be because most female animals are kept indoors for breeding and reproduction under good and clean management and hygienic conditions, whereas most male animals are left outdoors for grazing and therefore exposed more to infection. Seasonal variation was another significant risk factor associated with the occurrence of hemonchosis in goats, which has been documented in several studies (56). Additionally, this study showed a higher prevalence of infections among animals in hot seasons (22.22%) than in cold seasons (8.33%). A study in India has revealed that the prevalence of gastrointestinal parasites in goats was highest in monsoon (94.60%), moderate in summer (87.50%), and lowest in winter (63.15%) (57). In contrast, Yadav et al. (58) have revealed that the prevalence of gastrointestinal nematodes was recorded throughout the year, but it was higher during rainy seasons (88.54%), followed by summer (83.15%) and winter (76.01%). Another study has reported that worm burdens of hemonchosis were higher in wet seasons than in dry seasons (59). Moreover, it is possible that a combination of breed and environmental factors is responsible for this observation. The findings of this study confirmed the possible influences of seasonal variation on the occurrence of *Haemonchus* spp. in goats (57).

The control of hemonchosis mainly relies on the use of proper anthelmintics, which are necessary for effective treatment; however, there is a rising concern in the number of reports of drug resistance to these parasites (60). The diagnosis of the infection is critical for the implementation of effective intervention strategies. These parasites can be detected using different techniques, for example, the modified Baerman technique for detecting larvae and fecal egg count and the modified McMaster technique using flotation methods for detecting parasitic eggs (61, 62). However, the differentiation of *Haemonchus* eggs from other co-infection cases with other gastrointestinal nematodes remains difficult (63, 64). The identification and counting of larvae in fecal cultures can also be used, but this method is not readily applicable (63, 64). Clearly, more accurate methods are required for detecting such mixed cases of infection besides their importance in the differentiation of eggs of *Haemonchus* spp. from other gastrointestinal nematodes. Interestingly, using morphometric techniques and molecular-based detection methods, for example, polymerase chain reaction and loop-mediated isothermal amplification assays using an internal transcribed spacer (ITS-1), targeting rDNA regions, have been used and offered many advantages for detecting *Haemonchus* (63, 65–68). Despite this fact, the cost of these molecular-based methods represent one of the main barriers for their application at a large-scale level, particularly in developing countries (68).

Regarding the morphological characteristics of *Haemonchus* spp., they were characterized by the presence of four lancets in the anterior end and two opposite cervical spines. Males showed a well-developed bursa with an asymmetrically placed dorsal lobe, which is supported by a characteristic Y-shaped dorsal ray and a prominent vulvar flap, which showed both a thumb-like and reduced knob-like forms. These morphological characteristics are consistent with those reported in several studies that morphologically characterized *H. contortus* in goats (69–71). Importantly, the role played by SEM in detecting and characterizing *Haemonchus* spp. was documented in several studies (21, 72–76). In this study, SEM was used to identify and differentiate some ultrastructures that aid in the taxonomic differentiation of several *Haemonchus* spp. It has shown different shapes of vulvar flaps. As shown in the present results, the vulvar region of female *H. contortus* showed a thumb-like flap, whereas that of female *H. placei* exhibited a reduced knob-like flap. In contrast, the vulvar region of female *H. longistipes* showed a small rough nut-like vulvar flap. These findings on vulvar morphology are consistent with those reported in a previous study (20), showing that linguiform females predominate *H. contortus*, prominently knobbed females predominate *H. placei*, and small knobbed smooth females predominate *H. longistipes*. Another study (77) has documented the role of vulvar morphology in identifying *Haemonchus* spp. in goats, and the results showed that vulvar morphology was 53.8% linguiform, 18.5% knobbed, and 27.6% smooth. In the same study, different species of the parasite were identified based on morphometric parameters of the spicules of mature males of *Haemonchus* spp. in goats (77), whereas the identified species were as follows: 96.6% were *H. contortus*, 2.9% were *H. placei*, and 0.5% were *H. longistipes*.

Note that using the spicules might provide the easiest and quickest method for differentiating various species, for example, *H. contortus* and *H. placei*, identified by SEM (78, 79). In this study, the spicules of male *H. contortus* showed fused barbed tips, whereas that of male *H. placei* exhibited split barbed tips, which are the same features described elsewhere (1). The spicule length in this study was 364.5 μm in *H. contortus*, 326.5 μm in *H. placei*, and 280.5 μm in *H. longistipes*. A study involving cattle and sheep distinguished *H. similis, H. contortus*, and *H. placei* from each other based on the structure and length of the spicules, vulva, and tail length (1). In a study by Lichtenfels et al. (1), the average spicule length was 425 μm in *H. contortus*, 481 μm in *H. placei*, and 341 μm in *H. similis*. Importantly, the female tail length is also among the criteria of differentiation of the parasite species, namely, *H. placei* and *H. similis* (1). In this study, the average female tail length was 312.4 μm in *H. contortus* and 279 μm in *H. placei* with a dome-shaped anal pore, whereas the average tail length in *H. longistipes* was 179 μm with an elongated transverse slit. In a study, the authors have reported a short (135–278 μm) conical tail in *H. similis*, whereas the tail was long (378–720 μm) and almost filiform in *H. placei* (1). In contrast, female *H. contortus* had tails of intermediate lengths, measuring between those of *H. similis* and *H. placei* (1). However, it should be stressed that the financial constraints and need for infrastructure remain the major limitations of the use of SEM in routine examination, favoring the performance of other techniques, for example, molecular-based assays (13). The findings of this study revealed that SEM could help identify and characterize different *Haemonchus* spp. but is difficult to use in routine tests.

## Conclusions

Taken together, this study explored the role of morphological and morphometric methods in identifying and differentiating various *Haemonchus* spp. recovered from goats. The role of goats in the maintenance of the epidemiological foci of hemonchosis in Egypt has also been investigated. Furthermore, this study revealed the use of SEM in identifying three *Haemonchus* spp. recovered from goats in Egypt. This study recommends conducting further study to explore more about these species circulating in the Egyptian environment combined with confirmation and differentiation of the identified species of the parasite using molecular techniques. Furthermore, our data provides novel information which requires the attention of local authorities toward the application of more strict hygienic measures and implementation of effective control strategies against the unauthorized slaughtering of goats in private slaughter houses, which could be source of dozens of diseases of public health importance.

## Data Availability Statement

The original contributions presented in the study are included in the article/supplementary material, further inquiries can be directed to the corresponding author/s.

## Author Contributions

AG, RK, AD, MM, and MA were involved in the conception of the idea, developed the methodology design, and performed the data analysis and interpretation. NE, AT, DE-s, AR, EE, MK, KA, and EKE participated in the design of the methodology, sampling, laboratory work, and data analysis. EKE and AG contributed their scientific advice and prepared the manuscript for publication and revision. All authors read and approved the final manuscript.

## Funding

This work was supported by Taif University Researchers Supporting Program (project number: TURSP-2020/153), Taif University, Saudi Arabia.

## Conflict of Interest

The authors declare that the research was conducted in the absence of any commercial or financial relationships that could be construed as a potential conflict of interest.

## Publisher's Note

All claims expressed in this article are solely those of the authors and do not necessarily represent those of their affiliated organizations, or those of the publisher, the editors and the reviewers. Any product that may be evaluated in this article, or claim that may be made by its manufacturer, is not guaranteed or endorsed by the publisher.
